# Optimising Puppy Socialisation–Short- and Long-Term Effects of a Training Programme during the Early Socialisation Period

**DOI:** 10.3390/ani12223067

**Published:** 2022-11-08

**Authors:** Lisa Stolzlechner, Alina Bonorand, Stefanie Riemer

**Affiliations:** 1 Department of Cognitive Biology, University of Vienna, Djerassiplatz 1, 1030 Vienna, Austria; 2 Division of Animal Welfare, Vetsuisse Faculty, University of Bern, Länggassstrasse 120, 3012 Bern, Switzerland

**Keywords:** dog, *Canis familiaris*, puppy, socialisation, stress resilience, stress-coping, stress inoculation, startle recovery, novel object test, problem-solving task

## Abstract

**Simple Summary:**

The socialisation period in dog puppies (approx. 3–12 weeks) is one of the most important periods in determining later behaviour. Nonetheless, only a few studies investigated how socialisation can be optimised. Here, we tested whether providing small “challenge” exercises to puppies early in the socialisation period (between 3 and 6 weeks of age) enables puppies to cope better with stressful stimuli later. Eighty-three puppies from 12 litters of various breeds were enrolled. Half of each litter was assigned to the treatment group and was carefully exposed to potentially startling stimuli, novel objects, and problem-solving tasks over a period of three weeks. The other half of the litter served as a control group and received no additional exercises. All puppies were tested in a behaviour test at 6–7 weeks of age. Puppies from the treatment group were bolder towards a novel object, startled less after a loud noise, solved the problem-solving task faster, and showed higher persistence in problem-solving task than the control group. These findings demonstrate beneficial effects of the exercises. However, at the age of six months, there were no differences in owner-reported personality measures between the groups. To achieve lasting effects, it may be necessary to continue with the training exercises for a longer time period.

**Abstract:**

The socialisation period in dog puppies is one of the most important periods determining behavioural development in dogs. Here, we aimed to test the effect of providing stimulation (beyond mere exposure) early during the socialisation period (approx. 3–6 weeks) on puppies’ behaviour. Each of 12 litters (83 puppies) of various breeds was divided into a treatment and a control group. Between 3–6 weeks, the treatment group received age-appropriate “challenge” exercises (carefully graded noise exposure, novel objects, and problem-solving tasks) four times per week (total 12 times). The control group spent the same time with the trainer, who cuddled or played with the puppies. In a behaviour test at 6–7 weeks, two of four principal components, “social-startle” and “response to novelty”, differed significantly between the groups. Treatment puppies were bolder towards the novel object, showed a reduced startle reaction, and recovered more quickly after a loud noise. Furthermore, they accomplished the problem-solving task faster and were more persistent during problem-solving than the control group. The control group showed a higher interest in a friendly stranger. It is a possibility that increased handling experienced by the control group had beneficial effects on their sociability. No long-term effects of the treatment were found, as determined by a validated dog personality questionnaire, available for 67 dogs at the age of six months. Likely, a continuation of the treatment over a longer time period would be necessary to obtain lasting effects, since the training took place only during the first third of the socialisation period.

## 1. Introduction

Early life experiences are of prime importance in shaping later behaviour [1]. For instance, studies identified maternal care [2,3], early handling by humans [4,5,6,7,8], and environmental enrichment as important factors that affect animals’ behaviour and stress-coping ability in the long term [7,9,10,11,12,13]. Growing up in a stimulating environment is associated with better learning ability and memory, decreased fearfulness, and an improved ability to cope with acute stressors [14,15,16,17]. Environmental enrichment can be effective in preventing adverse effects from stress exposure as well as counteracting the effects of experienced stress [16].

There is evidence to suggest that the benefit of environmental enrichment lies in presenting low-level challenges, or stressors [18,19]. While chronic or severe stress has detrimental effects, exposure to mild and predictable stressors (such as exposure to novel objects) can improve an individual’s stress-coping ability. Careful exposure to mild stressors during sensitive periods early in life is known as “stress inoculation training” and has demonstrated beneficial effects on the development of arousal regulation and stress resilience in several species [18,20,21,22,23]. However, it is paramount to manage the intensity of any stressors presented carefully, since early life stress can have adverse consequences if its intensity is beyond the individual’s coping ability [23]. In human children, it has been suggested that frustration tolerance can be improved, and aggression reduced by presenting challenges and giving them opportunities to learn how to solve problems on their own [24]. In pet dogs, one small pilot study indicated that the provision of problem-solving games as mental/physical challenges has the potential to reduce fearfulness in adult dogs [25], but to our knowledge, this research topic has not yet been followed up.

In dogs, the socialisation period (3 to 12–14 weeks) is one of the most important periods determining later behaviour [26,27,28,29,30,31,32,33]. It commences at around three weeks of age when puppies’ eyes and ears become functional, and they become more mobile. Early in the socialisation period, puppies tend to fearlessly explore and investigate unfamiliar things in their environment, but they become increasingly wary of novelty with age [34,35]. From approximately three to six weeks, puppies exhibit a reflexive startle reaction (fast contraction of the muscles) in response to sudden/intense sounds [36], which is followed by immediate recovery. This behaviour is not comparable with an adult-like, active fear-related response, but is characterised by rapid habituation. The first fear responses occur around six to seven weeks, although this may vary by up to two weeks between different litters and breeds [36,37,38]. Thus, Lord [35] would argue that the socialisation period ends at around eight weeks, when puppies often show initial fear responses to novelty. However, this fear of novelty (leading to increased avoidance) increases further until 12–14 weeks, which other authors consider as the end of the socialisation period [26,31,37,39].

The ability to habituate to diverse stimuli without fear, or to overcome any fear quickly during the socialisation period, is key for dogs’ ability to cope with the diversity of stimuli encountered in a human world (c.f. [36,37,38]). Thus, it is important to expose puppies to a range of different stimuli such as noises, visual stimuli, and other sensory experiences as well as social interactions with humans, conspecifics and other species during this period of rapid neurological and emotional development [27,28,32,33,40,41].

Retrospective studies indicated strong associations between fearfulness and fewer experiences during the socialisation period (3 to 12–14 weeks) [27,28,29,30,32,33].

At no other life stage do dogs habituate as easily to novel and potentially startling stimuli as during this sensitive period [34,40,41], which takes place when the puppies are mostly still living with the breeder. As a consequence, interventions to improve socialisation carried out by breeders would have great potential to improve dog welfare on a large scale. Nonetheless, only a handful of studies have investigated interventions to optimise socialisation in puppies.

One study exposed 37 German shepherd puppies to radio programmes three times a day for 20 min during the ages of 16–32 days. In a behaviour test at seven weeks of age, puppies that had experienced previous noise exposure were rated as having more favourable responses to presentations of intense sudden noise [42]. However, in a different study, no beneficial effect of early auditory stimulation (presentation of radio, music or ambient noises during feeding) on fear responses to a loud noise at seven weeks were identified [43]. Instead, the authors found a positive effect on behavioural reaction to manipulations, different environmental stimuli and interaction with humans [44]; thus, the mechanism of the potential benefits of the auditory stimulation are not clear.

Vaterlaws-Whiteside and Hartmann [45] investigated an early socialisation program in six litters of retrievers (34 puppies) purpose-bred as guide dogs. The stimulation the puppies received under standard rearing procedures appears to have been relatively limited. Half of each litter received additional socialisation five times a week between the ages of one and six weeks, including presentation of tactile, auditory, and visual stimuli, as well as human interaction/handling and problem-solving tasks [45]. In a behaviour test encompassing eight stimuli ranging from engaging with a human to reacting to various stimuli and objects, the treatment group received better scores in responsiveness to a human assessor and environmental stimuli [45].

When the puppies were eight months old, their puppy walkers completed a dog personality questionnaire, which indicated more favourable scores for separation-related behaviour, distraction, general anxiety, and body sensitivity in the treatment group compared to the control group. Thus, a long-term beneficial effect of the additional stimulation was demonstrated [45].

Anecdotally, stress resilience (encompassing ability to both cope with acute stress and recover after stress exposure [44]) can be improved further by presenting challenge exercises (c.f. “stress inoculation”) to young puppies—thus going beyond mere stimulus presentation [46]. This is supported by a study indicating that vertical nursing (dams nursing their puppies while standing or sitting as opposed to lying down, making it more challenging for the puppies to suckle), was associated with a higher rate of certification in prospective guide dogs [47]. If breeders could (re)create some challenges for their puppies, this might have great potential to promote their stress-coping abilities.

To test this hypothesis, we performed a controlled study on the effect of age-appropriate “challenges” (exposure to potentially startling stimuli, novel objects, and problem-solving tasks) in the early socialisation period. We predicted that dogs that received such additional training as puppies would show less pronounced fear responses when exposed to novel environments, objects, and loud noises and/or recover more quickly, and show enhanced problem-solving abilities. We tested this prediction by performing a behaviour test with the puppies at the age of six to seven weeks. Additionally, when the puppies were six months old, their owners filled in a dog personality questionnaire. Personality traits relative to members of the same age cohort have been shown to be relatively stable by the age of 6 months in pet dogs [48,49]; therefore this was deemed an appropriate time point to measure longer-term effectiveness of the treatment.

## 2. Methods

### 2.1. Subjects

Subjects of this study were 83 puppies (*Canis familiaris*) of eight different breeds (Table S1). Small-scale dog breeders (with no more than two litters per year) in Eastern Austria were recruited via online advertisements (dog breed clubs, web sites, Facebook). All puppies were bred according to FCI (Féderation Cynologique Internationale) standards. Breeders were excluded from the study if they already provided very high levels of stimulation to their litters. Eleven breeders with twelve litters of four to eleven puppies were selected (for details, see Table S1). The puppies spent most of their time in the house. To minimise stress as well as hygiene risks, all training and testing took place at the breeders’ homes.

Each litter was divided into a treatment and a control group. Puppies were semi-randomly assigned such that the sexes were equally distributed between the two groups. Handling or feeding of the puppies by the experimenter (LS) was performed as similarly as possible in the two groups. For example, for some exercises, puppies in the treatment group were picked up or fed; on the corresponding day, puppies in the control group were also handled and fed. The experimenter spent approximately the same amount of time with both groups of puppies. While the puppies from the treatment group participated in the exercises, the experimenter petted or played with the control-group puppies. When the control-group puppies were sleeping, the experimenter remained neutral and watched them.

### 2.2. Treatment Phases

For each litter, the training of both groups started four to ten days after eye-opening, and was performed over the course of three weeks. We chose to end treatment at 5–6 weeks to be able to conduct the behaviour test at around six weeks, well before the fear period commenced at approximately eight weeks [32]. During this time, puppies were trained four times a week, in total, 12 times.

#### 2.2.1. Preparation Phase

The puppies were normally kept in their home room (hereafter, room A). Once the puppies had started to open their eyes (between two and three weeks of age, depending on the litter), both mother and puppies were habituated to a different room (hereafter, room B). This was done to prepare the puppies for separation into the two groups during the training exercises (so that only the treatment group was exposed to a stimulus). To this end, the breeder transferred the litter into room B by carrying them in a basket or individually. Depending on the set-up available at the breeder’s, the puppies were kept in a whelping box or puppy pen during this time. All breeders performed this training for two to four days before the experimenter became involved. Over several days, the breeder increased the time that mother and litter spent in room B (or in one case, the garden) to up to 30 to 60 min per day.

#### 2.2.2. Habituation Phase

Following the preparation phase, both the treatment group and the control group were habituated to being separated from the other half of the litter and the mother (but not to individual separation) on two consecutive days. In order to ensure that the study design was counterbalanced, in half the litters, the control group was taken to room B on the first day and remained in room A on the second day, and vice versa for the other half of the litters. The puppies’ mother was given free choice to join the puppies in room B, to remain with part of the litter in room A, or to spend time elsewhere, and this varied between bitches and days. The separated group remained in room B for 30 min, during which the experimenter (LS) stroked and talked to the puppies. During these first two days of separation, no exercises were presented, so that both groups had had the same experiences when the treatment phase started. We considered habituation successful when all puppies in the litter showed relaxed and calm behaviour in room B.

#### 2.2.3. Training Phase

Once the habituation phase was completed, the treatment phase commenced. The first author carried out all the training. On each day of training, the treatment group was presented with four exercises: one novel object, one problem-solving exercise, and two startle response and recovery exercises. The order of presentation of the different exercises was adjusted to the puppies’ activity level and so varied from day to day. In total, 12 different novel objects, 10 different problem-solving tasks (the shaping and detour challenges were presented twice at different levels of difficulty), and 24 different sounds were presented during the treatment phase (Table 1 and Table 2). A typical progression would be as follows: often puppies were sleeping in the beginning, and the experimenter performed the first startle and recovery exercise (often, the puppies continued to sleep during this). Afterwards, the problem-solving task was performed, upon which the puppies typically fell asleep again. The novel objects were either presented in the beginning or after the problem-solving task.

*Startle and recovery exercises – procedure*

A **noise stimulus** (e.g., dropping a heavy book at a distance of three meters from the puppies) was presented to all puppies of the treatment group together. With each litter, presentation started from a high distance and at low volume. Over time, the distance was decreased and the volume was increased (with approximately 5–30 s between presentations, depending on the puppies’ behaviour). The noise stimuli were expected to induce a slight reflexive startle response that should be followed by immediate recovery and resuming of the previous activity [36]. When the puppies showed no startle response at all to the presented stimuli, either the volume was increased or the distance to the puppies was decreased, taking care to adjust the levels of stimulation to the puppies’ behavioural reactions. Thus, depending on the puppies’ behaviour, the intensity of presented sounds differed between litters. Sometimes, puppies were sleeping deeply during the auditory stimulation, so that they did not show any visible reactions.

Recorded sounds were played back from a commercial loudspeaker (Bose^®^ SoundLink color II Bluetooth wireless speaker). A mobile phone app, “Schallpegelmesser in Dezibel”, was used to estimate the relative volume of the stimuli. While we did not calibrate the app before use, the phone used was always the same and the experimenter attempted to use a constant distance from the sound source. Thus, readings of volume cannot be considered accurate, but may allow some inferences regarding the relative loudness of the sounds presented.

The sounds were presented from a distance ranging from 4–323 cm from the puppy pen, and varied from 15–79 dB, as measured by the mobile phone app “Schallpegelmesser in Dezibel”. Objects were dropped from different heights (ranging from 5–70 cm) to create different intensities of the sound, depending on the stage in training (first or second noise exposure in the day, and first or second training session with the sound), as well as the noise sensitivity of the litter. At different times during the treatment day, this exercise was performed with two different noises.

On day three of the treatment, one puppy showed a stronger startle response on the first startle recovery exercise and did not return to its previous behaviour within 30 s. Training was terminated for the day for this puppy. On the next day, the puppy was not included in the group when the two startle and recovery exercises were conducted. Instead, the experimenter performed the sessions separately with the puppy, with a lower intensity exposure. From then on, the puppy was again included in the group exercises without any issues.

*Separation exercise* *– procedure*

During the **separation exercise**, one puppy was briefly separated from the rest of the treatment group by means of a barrier grid (the experimenter was present with the “separated” puppy throughout). The other puppies in the treatment group remained in the same room to minimise stress, and at the beginning of the treatment phase, they could see each other through the grid. Over several training sessions, the level of separation was increased from one to three minutes, and the degree of separation was heightened by gradually blocking the puppies’ view by placing a towel over the grid.

*Problem-solving task* *– procedure*

Once separated, each individual received an age-appropriate **problem-solving task** (duration: 1–5 min, depending on the type of task and the puppy’s success in solving the problem). For example, they had to traverse a barrier to reach a food reward (exercises adapted after [46], Table 1 and Table 2). The progression of tasks was such that easier tasks preceded more difficult tasks. If a litter was already familiar with an object used for a problem-solving task from their home environment, the experimenter chose a different problem-solving task. The following challenges were used as replacement challenges: find-food challenge, tea towel challenge 2, and detour challenge 3 (Table 1 and Table 2).

*Novel object task* *– procedure*

Thereafter, all puppies from the treatment group were placed together again, and when the puppies were in the pen again, **a novel object** (Table 1 and Table 2) was placed inside the pen (or, in case of the scooter, moved around in front of the pen). The experimenter touched and moved the object around, as well as holding it still to allow the puppies to explore it. If a litter was already familiar with an object from their home environment, the experimenter chose a different object to ensure novelty. The following items were used as replacement objects: a helmet, a backpack, a pump, an electric trimmer, and a metal bowl (Table 1 and Table 2). Exposure lasted between 1 and 10 min, depending on the type of object, the puppies’ behaviour, and risk of destruction of the object.

The order of exercises varied between litters, depending on the activity levels, familiarity with certain objects, and noise sensitivity observed in the litter. One training session for the entire treatment group lasted from 10 to 40 min (depending on the exercises, the age of the puppies, and the number of puppies in the litter), including breaks. The trainer alternated the days that she worked with the treatment and control groups, respectively, first. When the treatment group was trained first, the time spent with the puppies was matched in the control group. When the control group was trained first, the experimenter used the average time that was needed for the exercises one day before to spend with the control group.

### 2.3. Behavioural Testing

At the age of 6–7 weeks, all puppies were tested in a behaviour test (adapted from Riemer et al. [48]), which consisted of five subtests: exploration of an unfamiliar room, contact with a friendly unfamiliar person, a novel object, a problem-solving task, and exposure to a loud noise (see Table 3). All five subtests were performed in one session (approximately 20 min per puppy) in the order presented in Table 3. In four out of five subtests, three people (all females) were present during the tests: the breeder, the experimenter, and the camerawoman, who also served as “stranger” during the **greeting test** (second subtest). The stranger was previously unfamiliar to all puppies and was blind to their group assignment. During **room exploration** (first subtest), only the cameraperson was present, with the exception of two litters which behaved more anxiously than the other puppies. In these cases, for animal welfare reasons, the breeder remained in the room also during this subtest.

The **problem-solving task** (third subtest) consisted of two conditions: a solvable and an unsolvable condition. In the solvable condition, the puppy could obtain several pieces of food by knocking over a cup. In the unsolvable condition, the cup was attached to a piece of cardboard, so that the puppy was unable to move it.

During the **startle test** (fourth subtest), a balloon was burst approximately 3 m from the puppy. Two litters included puppies that were found to be more noise sensitive than the rest during treatment, and one litter was tested in a very small room so that a distance of three meters from the puppy could not be achieved. Therefore, instead of the burst balloon, these litters were presented with a different novel noise (either an eyeglasses case that was closed quickly (creating a snapping noise), or a plastic bowl that was dropped on the ground) with lower dB (for both of these stimuli, the average volume, according to the phone app, was 50 dB). As we tested the puppies within their sensitive period, we did not want to risk exposing them to stimuli that might be perceived as highly threatening.

In the fifth subtest (**novel object test**), the puppies were exposed to an unfamiliar battery-powered cat toy that performed erratic movements.

As the treatment groups were balanced within in each litter, we do not expect major influences of variation in the presentation of subtests 1 and 4 on treatment outcomes. Nonetheless, it cannot be ruled out that stronger treatment effects would emerge under more stressful conditions (i.e., exploration of an unfamiliar room without a familiar person present; louder noise).

#### 2.3.1. Behavioural Coding

The puppies’ behaviours in the test were videotaped and subsequently coded using Solomon Coder (© András Péter) by a blinded coder (AB), who was unaware of the details of the treatments and the group allocation. Most behaviours were coded as durations, such as time spent exploring, whimpering, being near a person, being in body contact with the stranger, touching the novel object, solving the problem-solving task, and touching the novel object, latency to solve the problem-solving task, and latency to touch the object of the problem-solving task. Ordinal scores were used for tail position during exploration, approach towards the unfamiliar person, puppies’ initial startle reaction to the noise stimulus, change in the activity of the puppy after the noise, and whether the puppy played within 15 s after the noise (Table 4).

#### 2.3.2. Inter-Rater Reliability

To assess inter-rater reliability of the behavioural codings, LS coded one randomly selected puppy from each breeder (total: 12 puppies = 15% of the videos). Reliability was adequate for all variables (Table 5 and Table 6).

### 2.4. Dog Personality Questionnaire

When the puppies were six months old, we sent out the German version of the Dog Personality Questionnaire (DPQ; short form) after Jones [50] (German version previously published and validated in Riemer, et al., [51] and Turcsán, et al., [52]) to the owners who had adopted the puppies. The questionnaire includes 45 descriptive questions, e.g., “Dog is relaxed when greeting people”, “Dog is curious”, etc., which in the German version are rated on a 5-point Likert scale. The questionnaire yields 5 personality factors (hereafter “DPQ factors”) subdivided into 15 facets, or sub-level traits ([50,51,52]; Appendix A). Additionally, since one focus of the study was on preventing noise sensitivities, owners were asked to rate their level of agreement with the statement “My dog is afraid of loud noises” on a 5-point Likert scale.

The dog personality questionnaire was returned for 67 dogs. 

### 2.5. Analysis

Statistical analyses were carried out using IBM SPSS Statistics Version 23 (IBM Corporation and its licensors 1989, 2015) [53] and R Version 3.6.1. (R Core Team A Language and Environment for Statistical Computing 2019, Vienna, Austria [54].

#### 2.5.1. Analysis of Behaviour Test Results

The variables from the behaviour test were reduced using a non-linear Principal Components Analysis [55], function CATPCA (categorical PCA) in SPSS, with Varimax rotation.

The effects of treatment, age at testing, sex, and litter size on the four ensuing principal components were investigated using linear mixed-effects models, with litter as random effect (function lme in R).

Model assumptions were checked by visual inspection of residual plots, and if necessary, the analysis was repeated with transformed variables. No transformation was needed for the components “Explore” and “Novel Object”, except that the “Novel Object” component was multiplied by −1 to facilitate interpretation of this component, such that higher values on this component reflected boldness, rather than fearfulness. The “Social-Startle” component and the “Whimper” component were transformed. First, a constant of 6 was added (to obtain all positive values enabling further transformation such as squaring). The ensuing result for “Social-Startle” was subsequently squared. The final dependent variable for “Whimper” was calculated by dividing 1 by the [value for “Whimper” plus 6]. Cohen’s d was calculated as a measure of effect size.

To assess the influence of litter, the model for each of the four principal components, with litter included as a random factor, was compared with the model without litter. If these differed significantly from each other, litter was retained in the final model. In the results, we report the *p*-value of the ANOVA comparing the model with and the model without litter to gauge the effect of litter.

#### 2.5.2. Analysis of Dog Personality Questionnaire Results

Due to the ordinal nature of the DPQ questionnaire factors, Mann–Whitney U tests were used to test for differences between the treatment and control group in these personality measures, as well as for differences in fear of loud noises (extra question).

The effect of litter on the dog personality questionnaire results was assessed using Kruskal–Wallis tests.

#### 2.5.3. Correction for Multiple Testing

The corrected alpha level according to sequential Bonferroni correction was calculated to determine whether the results were significant after correction for multiple testing. 

When correcting for multiple testing, we considered as families [56] (1) the four linear mixed-effect models assessing the effect of treatment on the four principal components from the behaviour test; (2) the six Mann–Whitney U tests assessing effects of treatment on the five DPQ factors plus the question on noise sensitivity; and (3) the six Kruskal–Wallis tests assessing effects of litter on the five DPQ factors plus the question on noise sensitivity. 

With one exception (as mentioned in the text), the results remained significant after correction. We report the original *p*-values in the results, alongside the corrected alpha levels after sequential Bonferroni correction. If all results were non-significant even before correction, we did not indicate the corrected alpha level.

## 3. Results

### 3.1. Behaviour Test Results

Based on the maximum number of components with acceptable internal consistency (Cronbach’s alpha > 0.6), the nonlinear PCA over the 19 behaviour variables yielded four principal components explaining 55.8% of variance (Table 7). We consider variables with loadings >0.3 as loading on a given component.

1. The first principal component explained 16.2% of the variance and was labelled “**PC 1—Social-Startle**”. The following variables had high positive loadings on this component: time spent in body contact with the stranger during the greeting test (both when ignored and when the stranger initiated the interaction; a short latency to approach the stranger in the greeting test; initial startle reaction after the loud noise [score]; and activity after the noise [score]. Latency to approach the novel object and time spent touching the problem-solving device loaded negatively on this component.
2. The second component, labelled “**PC 2—Whimpering**”, explained 14.5% of variance and had positive loadings for whimpering in all subtests where whimpering was measured.
3. The third component, labelled “**PC 3—Novel Object**”, explained 13.3% of variance and had positive loadings for the activity after the loud noise and latency to play after the loud noise, duration of whimpering, and the time spent near a person during the novel object test. Time spent touching the novel object and the novel object approach score loaded negatively on PC3.
4. The fourth component, labelled “**PC 4—Exploration**”, explained 11.8% of variance and had positive loadings for activity during exploration, time spent near the stranger, and tail position during the exploration test, and negative loadings for time spent whimpering during the unsolvable problem-solving task and the latency to solve the solvable problem (Table 7).

**Figure 1:**
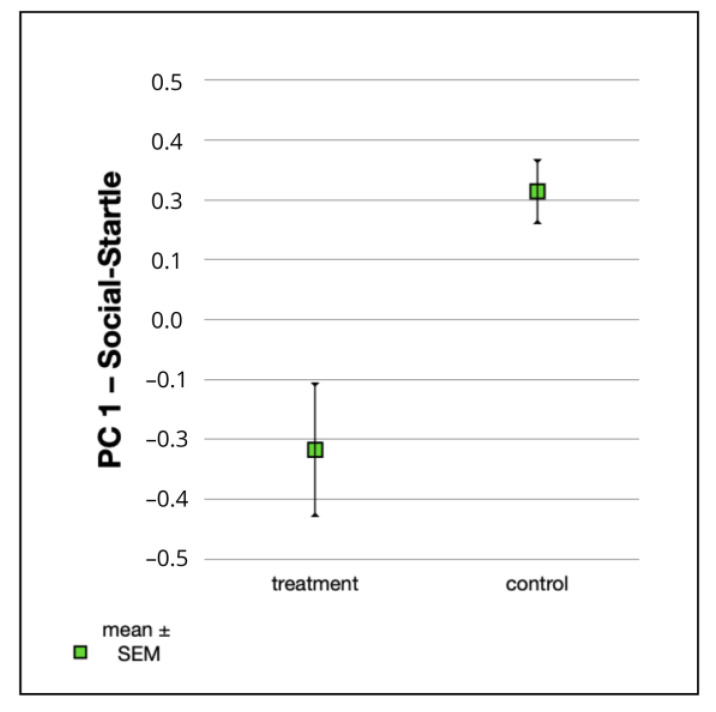
Mean values ± SEM for “PC 1—Social-Startle” in the treatment group (n = 42) and the control group (n = 41).

Linear mixed-effect models testing for the effects of treatment, age, litter and sex demonstrated a highly significant difference between the treatment group and the control group in “PC 1—Social-Startle” (Table 8) and in “PC 3—Novel Object” (Table 9). The treatment group had significantly lower values for “PC 1—Social-Startle” than the control group (F_1,70_ = 8.93, *p* = 0.0039) (Figure 1, Table 8). Puppies from the treatment group showed a less pronounced initial startle reaction and fewer fear-related activity changes after the loud noise than puppies from the control group. They had a longer latency to approach the stranger during the greeting test than the control group and spent less time in body contact with her, both when she ignored them and when she tried to initiate an interaction.

**Figure 2:**
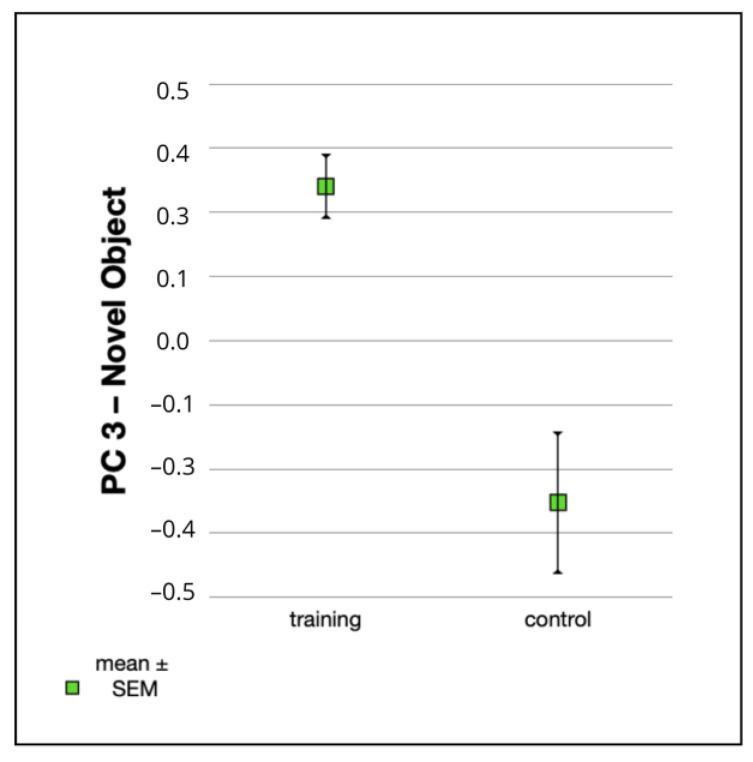
Mean values ± SEM for “PC 3—Novel Object” in the treatment group (n = 42) and the control group (n = 41).

The treatment group had a significantly higher value for “PC 3—Novel Object” (F_1,70_ = 8.75, *p* = 0.0042) than the control group (Figure 2, Table 9). The treatment group approached the novel object more quickly, spent more time in contact with the novel object, whimpered less, and spent less time near a person during the novel object test than the control group. Furthermore, treatment puppies resumed play more quickly after the loud noise and solved the solvable problem task with a shorter latency than the control group.

**Figure 3:**
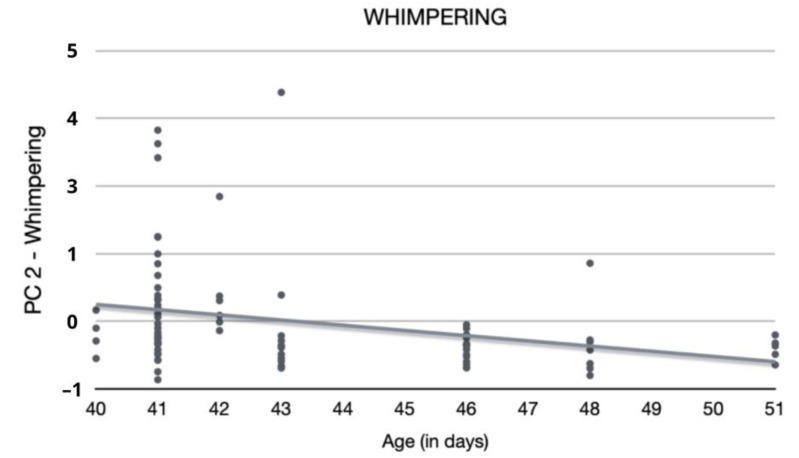
Association between puppies’ age at testing and “PC 2—Whimpering”.

There were no significant effects of treatment on “PC 2—Whimpering” (F_1_,_69_ = 1.7, *p* = 0.1962; Table 10) and “PC 4—Exploration” (F_1_,_69_ = 0.04, *p* = 0.8511; Table 11). Whimpering tended to decrease with age (Figure 3; Table 10). No other demographic variables were significantly related to any of the components.

The effect of litter was significant for “PC 1—Social-Startle” and “PC 3—Novel Object”, but not for “PC 2—Whimpering” and “PC 4—Exploration” (Table 8, Table 9, Table 10 and Table 11).

### 3.2. Dog Personality Questionnaire Results at the Age of 6 Months

There was no difference between the treatment group and the control group in personality factors derived from the Dog Personality Questionnaire, nor in owner-reported fear of loud noises (Table 12).

The results for litter effects on the questionnaire results are shown in Table 13. There were highly significant effects of litter on DPQ Factor 1, “Fearfulness”, and Factor 2, “Aggression towards people”. No litter effects were found for Factor 3, “Activity/excitability”, and Factor 4, “Training”. There was a trend for an effect of litter for Factor 5, “Aggression towards animals”. The effect of litter on fear of loud noises became marginally nonsignificant when applying sequential Bonferroni correction. 

## 4. Discussion

We investigated the short-term and long-term effects of “challenge exercises” (problem-solving games, novel objects, and potentially startling stimuli) during the early socialisation period on puppies’ behaviour. At the age of 6–7 weeks, the presentation of these challenges was associated with indicators of improved stress resilience. However, at the age of six months, there was no significant effect of treatment on owner-reported personality traits between the treatment and the control group. In contrast, significant effects of litter on several personality factors were found. This indicates that genetic effects and/or effects of the environment unique to each litter (independent of the training sessions) had a greater influence on behaviour at six months than the exercises between the 4th and 7th week of life. It is likely that a continuation of the treatment over a longer time period would be necessary to obtain lasting beneficial effects, since during the behaviour test at 6–7 weeks, the treatment group showed signs of higher boldness and better stress resilience than the control group in several contexts.

Puppies from the treatment group touched the novel object sooner and for a longer period of time, and they whimpered less than puppies from the control group during the novel object test. The latter spent more time in the proximity of a person during this test, which could be interpreted as seeking social support (c.f. [57]). Importantly, all of the puppies in the current study were well socialised by their breeders and had exposure to most of the enrichment items presented in the study by Vaterlaws-Whiteside and Hartmann [45]. Thus, the beneficial effect of the exercises presented to the treatment group on boldness go beyond those investigated by Vaterlaws-Whiteside and Hartmann [45].

In response to the startle stimulus (loud noise), treatment puppies showed a reduced startle response and faster recovery (lower latency to start playing) compared to the control puppies. This suggests that the repeated—but carefully controlled—exposure to startling stimuli during the early socialisation period had enabled them to habituate and to generalise to novel sounds. Nonetheless, the effect was not sustained, as treatment groups did not differ in owner-reported sound sensitivity at six months of age (but see below regarding limitations of using a questionnaire to assess behaviour). If preventive training over a longer time period (such as until the puppies are adopted) can be shown to reduce the risk of noise fears in dogs, this would have major implications for canine welfare: up to half the pet dog population are affected by noise fears [58,59,60], and fearfulness displayed at a young age has been shown to further increase as dogs mature [48,60].

Two previous studies have used presentations of sounds to young puppies. In Chaloupková, et al. [42], puppies from three litters received auditory stimulation (radio broadcasts including spoken-word programmes and music three times a day for 20 min) between 16 and 32 days. The control group comprised 18 puppies from 8 different litters. In a behaviour test at seven weeks of age, puppies from the treatment group showed more favourable reactions than the control group to different types of sudden loud noises, suggesting a beneficial effect of the stimulation [42]. Unfortunately, possible genetic effects were not controlled for in this study, since different litters made up the treatment and control groups.

Alves, et al. [43], exposed a treatment group (N = 21) to two hours of auditory stimulation per day, starting at three weeks of age, while a control group with no such stimulation included 46 puppies. The stimulation included commercial music and radio talk shows, as well as noises such as car noise, sirens, and gunshots, played at the volume of a conversation. From week five on, noises were presented at the natural volume of gunshots and police sirens, and this was paired with play and food. Contrary to the prediction, no difference in reactions to loud noises between the treatment groups could be discerned when the puppies were tested in a puppy test at seven weeks. However, the two groups differed in other subtests including social interactions and reactions to restraint, with the control group receiving higher (i.e., for police dogs, better) scores. The authors concluded that “a rich and varied environmental stimulation may be more important and that no sole source of stimulation is essential” [43].

Thus, while previous results regarding the benefits of auditory stimulation for young puppies have been inconclusive, our controlled method of presenting stimuli with increasing intensity was associated both with less intense immediate startle reactions and with faster recovery after a sudden loud noise in the puppies (even though this noise was louder than any of the noises used during treatment). Several explanations for the success of our method may apply: firstly, it is possible that real-life noises are more beneficial than recordings. For instance, it is known that in the context of treating noise fears, recordings may not be realistic enough. Acoustics are affected by the quality of the recording or the speakers and the setup of the room, and some noise-phobic dogs show no reaction to recordings [58,61,62].

Secondly, exposure in the current study was carefully controlled. At the discretion of the experimenter, presentation was performed in such a way as to allow immediate startle recovery, and intensity was increased only when the puppies no longer showed a reaction at all. Although Alves, et al. [43], presented environmental noises at a reduced volume during the first two weeks, it cannot be ruled out that the presentation at full volume was too much for some puppies and might have induced a sensitisation, rather than a desensitisation.

Thirdly, different litters were assigned to the treatment and the control group in the aforementioned studies, whereas we were able to control for genetic effects by assigning half of each litter to the treatment group and the other half to the control group. Thus, possible between-litter variation likely had less of an effect on the outcome than in the previous studies.

Despite the apparent benefit of the treatment when the puppies were tested at the ages of 6–7 weeks, no long-term effect of the treatment could be found. Notably, all puppies in our study were raised in a highly enriched environment during everyday life, which has been found to trump effects of specific treatments early during ontogeny also in previous studies [43,63]. In comparison, in Vaterlaws-Whiteside and Hartmann [45], the control group was raised in a relatively impoverished environment, and additionally, puppies’ keeping conditions were standardised. Lower genetic variation (all dogs in Vaterlaws-Whiteside and Hartmann [45] were purpose-bred retrievers) as well as lower environmental variation (breeding facilities as opposed to private breeders in our study) would further favour the detection of smaller effects than would be necessary in our study to reach statistical significance.

Perhaps most important to consider is that our treatment took place very early in the socialisation period—the puppies only had three weeks of treatment, and so after treatment was terminated, they experienced another six to eight weeks of the socialisation period (assuming that this period ends at 12 to 14 weeks [26,27,32,39,64]). Viewed from this angle, the lack of long-term effect may not be surprising, and future studies should assess the effects of continuation of “challenge” exercises over a longer time period, such as until puppies are rehomed at eight weeks or older.

The treatment and the control group differed in their responses to a friendly stranger during the behaviour test: on average, the control group approached her more quickly and spent more time interacting with her. While we did not code subtle behavioural signs to infer possible fear during this test, from personal observations, we would exclude that the lower interaction in the treatment group reflected fear-based avoidance.

We hypothesise that the differences might result from differential handling experiences between the treatment and control group, resulting in increased social attraction to an unfamiliar person in the control group. While the total time the experimenter spent with each group was the same, in the treatment group, she spent a proportion of this time preparing the exercises. Although the experimenter took care to perform picking up and feeding equally often in both groups, overall, the control group experienced more petting and playing with the experimenter than puppies from the treatment group, who were engaged in the exercises during this time. Future studies could include a control group with no interaction with the experimenter to prevent additional socialising effects, since strictly speaking, both groups in the current study received enrichment (social enrichment vs. environmental challenges).

If our hypothesis is confirmed, this would also be an important finding in relation to puppy socialisation during the COVID-19 pandemic, which made it less easy to invite a large number of visitors for puppy socialisation. The current results suggest beneficial effects of repeated visits even by the same person, if she interacts with the puppies in a pleasant way. In line with this, also in Vaterlaws-Whiteside and Hartmann [45], increased human interaction and handling by a trained researcher experienced by the treatment group was associated with better scores in responsiveness to a human assessor at six weeks. Moreover, Foyer, et al. [65], found that puppies from smaller litters scored higher on sociability as adults, with one explanation put forward being that each puppy might receive more human attention in smaller litters. In our study, there was no effect of litter size on sociability, possibly due to the large variety of breeds included.

No significant effects of treatment on the puppies’ behaviour during the exploration subtest (“PC 4—Exploration”) nor on whimpering (all subtests—“PC 2—Whimpering”) were found. Thus, it may particularly be the more stressful situations of the behaviour test where the effects of “stress inoculation training” become apparent. Whimpering is commonly interpreted as signifying distress or anxiety [66,67], as well as seeking attention [68]. In our study, whimpering formed a separate component and was not associated with the puppy test components encompassing reactions to startling stimuli, problem-solving ability, or sociability, nor with treatment group. In Simpson [69], multiple functions were attributed to whimpering/whining (the two are combined in the source): greeting, frustration, pain, attention seeking, and defence. Although there were consistent individual differences in the amount of whimpering in our study, as reflected by the fact that whimpering in all subtests loaded on a single component, the relevance of this behaviour is not clear in this context since the component was independent from the others.

After Fox ([64], as cited in Jensen [70]), whimpering (like crying and whining) is an infantile sound, and in line with this, this vocalisation decreased with age in the current study. This finding can be relevant regarding exact timings of future puppy tests, if whimpering is to be used as a variable of interest. It would not affect the current study results, however, since each litter included both treatment groups so that age was identical for the two treatment groups.

There were significant effects of litter on “PC 1—Social-Startle” and “PC 3—Novel Object”, whereas no litter effects were found for “PC 2—Whimpering” and “PC 4—Exploration”. In line with our results, Riemer et al. [48], who used a similar puppy test, report litter effects for greeting, interaction (behaviours which in our study loaded on “PC 1—Social-Startle”) and “low boldness” (c.f. our “PC 3—Novel Object”). Additionally, unlike in the current study, litter effects also manifested in exploration/inactivity (c.f. our “PC 4—Exploration”). In any case, the existence of litter effects on at least some of the test outcomes at 6–7 weeks of age indicates the role of genetic and maternal effects and/or the shared early environment on behaviour during the test.

Our long-term results also show highly significant effects of litter on the factors “Fearfulness” and “Aggression towards people”. The effect of litter on noise sensitivity became non-significant after correcting for multiple testing. No litter effects were found for “Aggression towards animals” (although there was a trend before correction), “Activity/excitability” and “Trainability”. It is possible that the latter two factors were more affected by individual training differences than the prior factors. The existence of litter effects, but not treatment effects, at six months reinforces the importance of genetic factors and/or early environmental and maternal influences for puppies’ development beyond the specific training programme. The time-limited exposure to challenge exercises over three weeks early in the socialisation period in the treatment group was not sufficient to create lasting measurable effects.

### Limitations

The methodology employed has some limitations. The enrolled puppies belonged to various breeds and originated from different breeders; thus, there was significant variation in experiences during rearing (although too much stimulation provided by the breeder was an exclusion criterion for participation). Moreover, for reasons of puppy welfare, the treatment was not perfectly standardised. As puppies sleep a lot at this age, the order of the exercise types (novel object; problem solving task; startle recovery) within a day was adjusted to puppies’ activity level. For animal welfare reasons, we decided against certain interventions, e.g., waking puppies up to participate in a particular exercise at a particular time.

There was slight variation in the exercises presented to the different litters. The experimenter adapted the presentation of novel objects and problem-solving tasks for each litter a little, because some litters had previously been exposed to some objects in their home environment. Future follow-up studies would benefit from a more structured approach, in which puppies’ experiences (apart from the treatment) are more standardised, and the types of exercises and their order of presentation is identical for all litters (even if timing of presentation during the day may need to be adapted to activity level).

One puppy was excluded from the treatment group for two startle and recovery exercise sessions (both in the same day), having shown an enhanced reaction previously. After participating in two sessions with lower intensity stimuli, separate from the group, from the next day on, the puppy was successfully trained with the rest of the group again. We retained this puppy in the analysis so as not to bias the data (as removing less resilient individuals from the treatment group would exaggerate the measured effect of the treatment).

In the behaviour test, the first subtest (room exploration) was adapted for two litters and the breeder was present for these, but not for the remaining litters. Again, this decision was made for animal welfare reasons, as these litters had showed a higher tendency for fearful behaviour.

Furthermore, three litters were tested, not with the burst balloon, but with a lower intensity sound as startling stimulus, to accommodate either notable sound sensitivity in the whole litter or the setup of the testing room. We would expect treatment effects to become especially apparent under stressful conditions; thus, we could say that we were working against our own hypothesis by reducing the intensity of stimulation for some litters. Despite this, we found a significant difference between the treatment groups both in immediate reactions to the loud noise and in recovery. Therefore, we feel that the methodology was suitable to demonstrate the predicted effect of the treatment on noise sensitivity. On the other hand, no treatment effects were found for the exploration component (where 2 of 12 litters were tested with the breeder present). It cannot be ruled out that effects would have been stronger under more stressful conditions.

Finally, the behavioural assessment at six months was questionnaire-based. We used a validated personality questionnaire; however, this questionnaire targeted the traits of interest less specifically than in the behaviour test. It is a possibility that we would have seen group differences had we performed a similar behaviour test with novel objects, problem-solving tasks and startling stimuli at six months. It is a limitation of a questionnaire-based study that each dog is assessed by a different individual, and the owners may be biased to an extent. Thus, the standardised behaviour test is likely to be more sensitive to differences in reactivity to sounds than the questionnaire, and future studies should re-test dogs in a behaviour test as juveniles or (ideally) adults.

## 5. Conclusions

We conclude that controlled early exposure to a variety of age-appropriate exercises and controlled exposure to noises and novel objects has a positive influence on stress-coping ability in six-to-seven-week-old puppies. However, no long-term effects of this early-age training programme were found, possibly due to the short duration of the training programme, which took place only during the first third of the socialisation period. Further research is needed to investigate how continuation of such training can lead to the development of better stress resilience and consequently prevent future problem behaviour in dogs.

## Figures and Tables

**Table 1:** Selection of exercises presented in each week during the three-week treatment phase (a total of 12 sessions per dog). Each session included presentation of a novel object (while puppies were inside the pen), a problem-solving task (easier tasks were presented first), and two different sounds. If a litter was already familiar with a given object, a “replacement novel object” or “replacement problem-solving task” was presented instead.

Exercises	3–4 Weeks	4–5 Weeks	5–6 Weeks
**Novel objects**	Puffed-up plastic bag	Paper bag	Roll-down blind
	Umbrella	Plastic cone	Plastic bag
	Cat toy	Flamingo	Scooter
	Water bottle	Crumpled up paper	Mirror
**Replacement novel objects**	Helmet	Pump	Metal bowl
	Backpack	Trimmer	
**Problem-solving tasks**	Book challenge	Traversal challenge 1	Detour challenge 1
	Plate challenge	Mirror challenge	Detour challenge 2
	Wobble challenge	Barrier challenge	Shaping challenge 1
	Tea towel challenge	Traversal challenge 2	Shaping challenge 2
**Replacement problem-solving tasks**	Find-food challenge	Tea towel challenge 2	Detour challenge 3
**Startle** **&** **recovery exercises**	Book dropped	Cake tin dropped	Firework recording
	Kitchen pod lid banged	Eyeglasses case dropped	Metal kitchen pod dropped
	Kitchen pod lid dropped	Bicycle horn tooted	Metal food bowl dropped
	Cutlery dropped	Metal box with cutlery shaken	Metal clicker
	Wooden spoon drummed	Gunshot recording	Experimenter screamed
	Plastic bottle dropped	Hair dryer	Brush dropped
	Door slammed	Roll-down blind dropped	Plastic cone dropped
	Clapping hands	Umbrella dropped	Plastic bowl dropped

**Table 2:** Detailed descriptions of the exercises.

Exercise	Description
Novel Object	
Puffed-up plastic bag	An empty plastic bin bag (40 × 25 cm) was inflated by mouth, turning it into a balloon-like object. Inside the puppy pen, the experimenter touched this object and slowly moved it around, as well as holding it still to allow the puppies to explore.
Umbrella	An umbrella was opened and closed several times next to the puppy pen. Subsequently, the open umbrella was placed into the puppy pen.
Cat toy	A cat toy (25 × 7 cm) was placed inside the puppy pen. The toy had a weighted lower half, an internal bell, and a feather on the top. It wobbled, without falling over, when the experimenter tapped it or when the puppies grabbed the feather.
Water bottle	A 1.5-L plastic bottle, filled with water, was moved around, shaken, and knocked over in the vicinity of the puppies. Then, the experimenter left the bottle standing or lying for the puppies to explore.
Paper bag	An empty paper carrier bag (32 × 17 × 44 cm) was placed inside the puppy pen and moved around by the experimenter.
Plastic cone	A red plastic cone (17.5 × 12 cm) was placed into the puppy pen.
Flamingo	An inflatable plastic flamingo (18 × 11 cm) was placed into the puppy pen and was moved around by the experimenter. Before the puppies could bite it, it was removed from the puppy pen.
Crumpled up paper	Several pieces of commercial white printer paper (A4) were crumpled up and thrown into the puppy pen, one after the other.
Roll-down blind	Inside the puppy pen, the experimenter repeatedly extended and retracted a roll-down blind (55 × 55 cm).
Plastic bag	A sturdy empty plastic carrier bag (40 × 20 × 45 cm) was placed inside the puppy pen and moved around by the experimenter.
Scooter	A scooter (HUDORA 14708 BigWheel 205) was placed in front of or into the puppy pen. The experimenter was either riding it or moved it by hand, depending on the available space.
Mirror	A mirror (40 × 80 cm) was either placed into the pen against a wall or on the floor.
Helmet	A bicycle helmet was presented to the puppies on the experimenter’s head.
Backpack	A backpack (29.2 × 18.3 × 39.6 cm) from Amazon Basics was placed inside the puppy pen.
Pump	A bicycle pump was placed into the puppy pen.
Trimmer	An electric trimmer was turned on and off by the experimenter (CAMRY CR-2821 Haarschneider für Haustiere).
Metal bowl	A metal food bowl weighing 260 g with a diameter of 20 cm placed inside the puppy pen.
**Problem-Solving**	
Book challenge	Puppy was placed onto a book (3 cm high; 17.5 × 25 cm) and had to step down on its own.
Plate challenge	Puppy was placed onto a cool ceramic dinner plate, 25 cm in diameter (approx. 2 cm off the floor), and had to step down. The plate had been kept in the fridge for approximately 10 min.
Wobble challenge	Puppy was placed onto an inflatable wobble cushion (34 cm in diameter; 4 cm high) and had to step down.
Tea towel challenge	A tea towel (approx. 40 × 30 cm) was placed over part of the puppy’s body, so that the puppy had to wriggle out.
Traversal challenge 1	Puppy had to traverse an empty plastic carrier bag (40 × 20 × 45 cm), lying flat on the ground, to reach a human or their littermates.
Mirror challenge	Puppy had to traverse a mirror (40 × 80 cm) that was placed flat on the ground to reach a human or food.
Barrier challenge	Puppies had to climb over a metal grid barrier (10–16 cm high, depending on the size of the puppies) to reach littermates, human or food. To make the metal more comfortable for the puppies to climb over, it was covered by a towel.
Traversal challenge 2	Puppy had to traverse an empty paper carrier bag (32 × 17 × 44 cm), lying flat on the ground, to reach food.
Detour challenge 1	A bowl filled with food was placed behind a metal grid barrier. Puppies had to move around the grid in order to reach the food. The level of difficulty was adjusted to the litter and the individual. The food was placed 2–20 cm from the edge of the grid, and grid shape varied from a V-shape to a straight line.
Detour challenge 2	Procedure as in detour challenge 1—with a small increase of difficulty by increasing the distance the puppy had to detour.
Shaping challenge 1	A mat was placed on the ground. Each time the puppy touched the mat, the trainer marked the behaviour with a clicker and gave the puppy a food reward. All movements towards the ground—sitting, sniffing, lying down, etc., were clicked and reinforced.
Shaping challenge 2	Shaping exercise part 2 (continuation of shaping the puppy to approach and lie down on the mat).
Find-food challenge	Small pieces of food were placed under a tea towel (approx. 40 × 30 cm), and the puppy had to find them.
Tea towel challenge 2	A tea towel (approx. 40 × 30 cm) was placed over the puppy’s body, so that the puppy had to wriggle out, with increased difficulty (towel was covering the whole body).
Detour challenge 3	Detour challenge 3 (procedure as in detour challenges 1 and 2—with a major increase of difficulty by increasing the distance the puppy had to detour).
**Startle and recovery exercises**	
Book dropped	A book, weighing 1.3 kg, was dropped onto a tiled floor.
Kitchen pod lid banged	A kitchen pot lid, weighing 314 g with a diameter of 22 cm, was banged against a metal kitchen pot, weighing 1080 g with a diameter of 22 cm and a height of 17 cm.
Kitchen pod lid dropped	A kitchen pot lid, weighing 314 g with a diameter of 22 cm, was dropped on a tiled floor.
Cutlery dropped	Several pieces of cutlery were dropped into a metal box (14 × 10 × 18 cm).
Wooden spoon drummed	The experimenter drummed a wooden spoon (30 cm) against a metal kitchen pot, weighing 1080 g, with a diameter of 22 and a height of 17 cm.
Plastic bottle dropped	A 1.5-L plastic bottle filled with water was dropped onto a tiled floor from a height of 33 cm.
Door slammed	A door was slammed next to the puppy pen.
Clapping hands	The experimenter clapped her hands several times.
Cake tin dropped	A cake tin, weighing 314 g with a diameter of 27 cm, was dropped onto a tiled floor.
Eyeglasses case dropped	An eyeglasses case was dropped onto the floor.
Bicycle horn tooted	A bicycle horn, made of metal with a rubber air bag, was tooted (18.5 × 5 cm).
Metal box with cutlery shaken	A metal box (14 × 10 × 18 cm) with several pieces of cutlery inside was shaken.
Gunshot recording	A gunshot recording from YouTube was presented to the puppies via a loudspeaker (Bose^®^ SoundLink color II Bluetooth wireless speaker). https://www.youtube.com/watch?v=AGVz9zoWNrQ (accessed on 7 May 2018)
Hair dryer	A hair dryer was turned on and off several times.
Roll-down blind dropped	A roll-down blind was dropped onto a tiled floor.
Umbrella dropped	An umbrella was dropped onto a tiled floor.
Firework recording	A firework recording from YouTube was presented to the puppies via a loudspeaker (Bose^®^ SoundLink color II Bluetooth wireless speaker). https://www.youtube.com/watch?v=H7FANXaanG4 (accessed on 10 May 2018)
Metal kitchen pod dropped	A metal kitchen pot, with a diameter of 22 and a height of 17 cm, weighing 1080 g, was dropped onto a tiled floor.
Metal food bowl dropped	A metal food bowl, weighing 260 g with a diameter of 20 cm, was dropped onto a tiled floor.
Metal clicker clicked	A metal clicker (sold as a dog training tool; 10.4 × 6 × 2.4 cm) was clicked several times.
Experimenter screamed	The experimenter screamed for about 2 s with a high voice.
Brush dropped	A brush, with a diameter of 15 cm, was dropped onto a tiled floor.
Plastic cone dropped	A plastic cone (17.5 × 12 cm) was dropped onto a tiled floor.
Plastic bowl dropped	A plastic bowl (26 × 17 × 33) was dropped onto a tiled floor.

**Table 3:** Behaviour test at six to seven weeks of age (40–51 days).

Nr.	Subtest	Description	Duration
1.	Room exploration	The puppy was allowed to explore the unfamiliar room for 1 min. Except for two litters where the breeder remained in the room, only the unfamiliar camerawoman was present in the room.	60 s
2.	Greeting Test	The camerawoman from subtest 1 sat on the floor and stayed neutral in a predefined position for 30 s without making noises or movements. She sat cross-legged and did not move her hands or head, ignoring the puppy. After 30 s, she tried to initiate contact for 5 s by calling the puppy’s name, chatting in a friendly voice or clicking her tongue. When the puppy approached, she petted the puppy and talked to her/him in a friendly way. After 5 s of attracting the puppy’s attention and/or petting it, she behaved neutrally for 5 s, then initiated contact again. The 5-s sequences of interaction and ignoring were alternated until the end of the subtest.	90 s
3.	Problem-solving	A paper cup with small holes 1 cm in diameter, so that the puppy could smell the food underneath, was used. First, the cup was placed onto a piece of cardboard (20 × 30 cm) over several pieces of food (depending on the puppies’ diet), in view of the puppy. The puppy could obtain the food by knocking over the cup (solvable condition). Second, the experimenter followed the same procedure, but attached the cup to the piece of cardboard, so that the puppy was unable to move it (unsolvable condition). Each subtest (solvable and unsolvable) lasted 2 min.	120 s (solvable) and 120 s (unsolvable)
4.	Startle Test	A balloon was burst approximately 3 m away from the puppy. The breeder remained approximately 1 m from the puppy to be able to provide social support and tried to engage the puppy in play after the noise. The average sound intensity of the bursting balloon, as measured by phone app, was 90 dB. This was louder than any noise the experimenter had presented to the puppies before *.	60 s
5.	Novel Object	A battery-powered toy resembling a paper bag, approx. 20 × 10 × 5 cm, was placed in a predefined position 2 m from the puppy’s starting point. The object was performing erratic movements while remaining in one place.	120 s

* Three litters were tested with novel sounds of lower volume, as explained above.

**Table 4:** Definitions and variable type (score/duration) of behaviours coded from the behaviour test.

Variable	Variable Type	Possible Values	Description
Exploration			
Tail	Score (point sampling, every 30 s)	3	Tail mostly high: tail is held above the tail base.
		2	Tail mostly medium: tail is in line with the tail base.
		1	Tail mostly low: tail is lower than the tail base.
Active	Duration		Puppy is moving or exploring. Moving is defined as a forward or backwards movement; coding starts when dog starts to move leg, followed by body movement. Does not include if dog moves legs but does not change spatial position. Exploring is coded when the puppy’s nose is <5 cm from ground or from objects, apparently sniffing, mouthing, manipulating, or scratching objects with the paw.
Whimper	Duration		Puppy is producing a high-pitched noise.
Near Stranger	Duration		Puppy’s head is <50 cm from the stranger.
Out of Sight	Duration		Puppy is out of sight.
Greeting			
Greeting Approach Score	Score	0	Does not approach the stranger (10 cm from stranger’s hands) within 45 s after she started to attract the puppy’s attention.
		1	Approaches the stranger within 21–45 s after she started to attract the puppy’s attention.
		2	Approaches the stranger within 11–20 s after she started to attract the puppy’s attention.
		3	Approaches the stranger within 10 s after she started to attract the puppy’s attention.
		4	Puppy is already in contact with stranger when she starts to attract the puppy’s attention.
Whimper	Duration		Puppy is producing a high-pitched noise.
Near Stranger	Duration		Puppy’s head is <50 cm from the stranger.
Direct Body Contact	Duration		Direct body contact with stranger (only when puppy initiates contact).
Out of Sight	Duration		Puppy is out of sight.
Solvable problem			
Whimper	Duration		Puppy is producing a high-pitched noise.
Invisible	Duration		Puppy is out of sight.
Problem-solving Latency	Duration		The time from release of the puppy until the puppy starts consuming the food.
Unsolvable problem			
Whimper	Duration		Puppy is producing a high-pitched noise.
Touch Object	Duration		Puppy touches cardboard or paper cup with a body part. If puppy is touching object AND near a person, touch object was coded.
Invisible	Duration		Puppy is out of sight.
Near Breeder	Duration		Puppy’s head is <10 cm away from breeder. (In the analysis, this variable was added up with “Near Experimenter” and “Near Stranger” as “Near Person”).
Near Experimenter	Duration		Puppy’s head is <10 cm away from experimenter. (In the analysis, this variable was added up with “Near Breeder” and “Near Stranger” as “Near Person”).
Near Stranger	Duration		Puppy’s head is <10 cm away from stranger. (In the analysis, this variable was added up with “Near Experimenter” and “Near Stranger” as “Near Person”).
Novel object			
Novel Object Approach Score	Score	2	Approaches to within 20 cm of the novel object within 5 s.
		1	Approaches to within 20 cm of the novel object after 5 s.
		0	Does not approach the novel object to within 20 cm within 30 s.
Touch Object	Duration		Puppy touches novel object with a body part.
Out of Sight	Duration		Puppy is out of sight.
Whimper	Duration		Puppy is producing a high-pitched noise.
Near Breeder	Duration		Puppy’s head is <10 cm from Breeder. (In the analysis, this variable was added up with “Near Experimenter” and “Near Stranger” as “Near Person”).
Near Experimenter	Duration		Puppy’s head is <10 cm from Experimenter. (In the analysis, this variable was added up with “Near Breeder” and “Near Stranger” as “Near Person”).
Near Stranger	Duration		Puppy’s head is <10 cm from Stranger. (In the analysis, this variable was added up with “Near Breeder” and “Near Experimenter” as “Near Person”).
Startle			
Initial startle reaction (addresses how strong puppies’ first reaction was after the noise)	Score	0	No visible reaction.
		1	Weak reaction: only one body part moves (e.g., either ears or head).
		2	Strong reaction: two or more body parts are moving and changing position (e.g., head and paws).
		3	Very strong reaction: Puppy lowers body completely, their belly touches the floor OR puppy makes a sudden move with all body parts.
Activity after noise (addresses puppies’ further reactions after the startle stimulus)	Summary score with 1 point each for the below variables	0	Puppy does not change activity and keeps doing what s/he was doing before or runs towards noise.
		1	Puppy changes activity and does not run towards noise.
		1	Freeze (puppy stops movement for more than 2 s). Only codable if dog was moving at the time of the noise (otherwise coded as NA).
		1	Flee (puppy runs away from the direction of the noise).
		1	Seeks comfort from breeder (puppy hides behind the breeder or jumps up at her).
		1	Tail lowered for at least 2 s after noise.
Play after noise (addresses how fast puppies were playing after the loud noise)	Score	0	Puppy engages in play within 15 s after the noise.
		1	Puppy does not play within 15 s after the noise.

**Table 5:** Inter-rater reliability for durations.

Variables	Standardised Cronbach’s Alpha
Explore—activity	0.92
Explore—whimper	1
Explore—near stranger	0.67
Greeting test—body contact/ignored	0.99
Greeting test—body contact/interaction	0.99
Greeting test—whimper/ignored	No variance
Novel object—whimper	0.99
Touch novel object	1
Novel object—near person	0.84
Problem solving—whimper	No variance
Problem solving latency	0.99
Problem solving—whimper (unsolvable)	0.83
Problem solving—Touch object (unsolvable)	0.99

**Table 6:** Inter-rater reliability for scores.

Variables	Weighted Cohen’s Kappa
Explore—mean tail score	0.7
Greeting—approach score	0.7
Novel object—approach score	0.88
Startle—initial startle reaction	0.67
Startle—activity after noise	0.64
Startle—play after noise	1

**Table 7:** Components and component loadings of the CATPCA (Varimax rotation); variable loadings >0.3 are bolded.

Nr.	Variables	Explanation	PC 1— Social-Startle	PC 2—Whimpering	PC 3—Novel Object	PC 4—Exploration	Total
**1**	Exploration—whimper	Higher score = longer duration of whimpering	0.216	**0.383**	0.177	**−** **0.567**	
**2**	Exploration—near stranger	Higher score = longer duration near stranger	0.141	−0.165	0.026	**0.575**	
**3**	Exploration—tail position	Higher score = higher tail position	−0.059	0.176	0.077	**0.694**	
**4**	Exploration—activity	Higher score = more activity	0.117	0.127	−0.120	**0.850**	
**5**	Greeting test—whimper/ignored	Higher score = longer duration of whimpering while person ignored the puppy	0.053	**0.832**	−0.072	−0.131	
**6**	Greeting test—body contact/ignored	Higher score = longer duration of body contact with person who was ignoring the puppy	**0.875**	0.137	−0.091	0.090	
**7**	Greeting test—whimper/interaction	Higher score = longer duration of whimpering while person interacted with the puppy	−0.091	**0.879**	0.032	0.044	
**8**	Greeting test—body contact/interaction	Higher score = longer duration of body contact while person interacted with the puppy	**0.845**	−0.155	−0.071	0.103	
**9**	Greeting test—person approach score	Higher score = faster approach	**0.840**	0.132	−0.060	0.099	
**10**	Problem solving latency	Higher score = longer latency	−0.037	−0.100	**0.313**	**−** **0.469**	
**11**	Problem solving—whimper	Higher score = longer duration of whimpering	−0.023	**0.675**	0.103	0.178	
**12**	Problem solving—touch object (unsolvable)	Higher score = longer duration of touching object	**−** **0.366**	−0.151	−0.068	0.134	
**13**	Startle stimulus—initial startle reaction	Higher score = more fear indicators	**0.496**	−0.145	0.012	0.059	
**14**	Startle stimulus—play after noise	Higher score = longer latency to play	0.117	−0.248	**0.615**	−0.086	
**15**	Startle stimulus—activity after noise	Higher score = more fear indicators	**0.464**	−0.176	**0.377**	−0.242	
**16**	Novel object—touch novel object	Higher score = longer duration of touching novel object	0.178	−0.263	**−** **0.744**	0.005	
**17**	Novel Object—whimper	Higher score = longer duration of whimpering	0.172	**0.556**	**0.581**	−0.029	
**18**	Novel Object—near person	Higher score = longer duration of time near person (<10 cm away)	0.111	0.045	**0.699**	−0.075	
**19**	Novel Object approach score	Higher score = faster approach	**0.350**	−0.135	**−** **0.661**	0.037	
	**Cronbach** **’** **s alpha**		0.691	0.674	0.66	0.602	0.956
	**Eigenvalue**		3.086	2.746	2.527	2.251	10.61
	**Variance explained**		0.162	0.145	0.133	0.118	0.558

**Table 8:** Results of the linear mixed-effects model assessing the effect of treatment, age, litter size, and sex on “PC 1—Social-Startle”. The *p*-value for litter refers to the ANOVA comparing the models with and without litter included. “Bonferroni-corrected α for treatment” indicates the alpha level (after sequential Bonferroni correction) to determine whether the treatment effect was significant.

Predictor	Value	Std. Error	CI −95%	CI +95%	Cohen’s D	numDF	denDF	F	*p*	Bonferroni- Corrected Alpha
Treatment	−0.49	1.64	−8.15	−1.59	−0.71	1	69	−2.96	0.0042	0.0125
Age	−0.63	0.48	−1.72	0.46	−0.87	1	9	−1.30	0.2245	
Litter size	1.29	0.81	−0.53	3.11	1.06	1	9	1.6	0.1443	
Sex	0.59	1.75	−2.89	4.08	0.08	1	69	0.34	0.7342	
Litter									0.0113	

**Table 9:** Results of the linear mixed-effects model assessing the effect of treatment, age, litter size, and sex on “PC 3—Novel Object”. The *p*-value for litter refers to the ANOVA comparing the models with and without litter included. “Bonferroni-corrected α for treatment” indicates the alpha level (after sequential Bonferroni correction) to determine whether the treatment effect was significant.

Predictor	Value	Std. Error	CI −95%	CI +95%	Cohen’s D	numDF	denDF	F	*p*	Bonferroni-Corrected α for Treatment
Treatment	0.58	0.19	0.19	0.97	0.72	1	69	2.98	0.0039	0.0167
Age	−0.02	0.05	−0.14	0.1	−0.24	1	9	−0.36	0.7250	
Litter size	−0.09	0.09	−0.29	0.1	−0.71	1	9	−1.06	0.3140	
Sex	−0.27	0.21	−0.68	0.15	−0.31	1	69	−1.29	0.2017	
Litter									0.0191	

**Table 10:** Results of the linear mixed-effects model assessing the effect of treatment, age, litter size, and sex on “PC 2—Whimpering”. The *p*-value for litter refers to the ANOVA comparing the models with and without litter included. “Bonferroni-corrected α for treatment” indicates the alpha level (after sequential Bonferroni correction) to determine whether the treatment effect was significant.

Predictor	Value	Std. Error	CI −95%	CI +95%	Cohen’s D	numDF	denDF	F	*p*	Bonferroni-Corrected α for Treatment
Treatment	0.006	0.005	−0.68	0.15	−0.3	1	69	1.28	0.2040	0.025
Age	0.002	0.001	−0.2	−0.001	−1.53	1	9	2.19	0.0566	
Litter size	0.0009	0.002	−0.23	0.11	−0.56	1	9	0.5	0.6305	
Sex	0.003	0.004	−0.5	0.37	−0.07	1	69	0.55	0.5820	
Litter									0.1575	

**Table 11:** Results of the linear mixed-effects model assessing the effect of treatment, age, litter size, and sex on “PC 4—Exploration”. The *p*-value for litter refers to the ANOVA comparing the models with and without litter included. “Bonferroni-corrected α for treatment” indicates the alpha level (after sequential Bonferroni correction) to determine whether the treatment effect was significant.

Predictor	Value	Std. Error	CI −95%	CI +95%	Cohen’s D	numDF	denDF	F	*p*	Bonferroni-Corrected α for Treatment
Treatment	0.04	0.21	−0.38	0.46	0.05	1	69	0.2	0.8414	0.05
Litter size	−0.1	0.08	−0.28	0.09	−0.8	1	9	−1.2	0.2593	
Age	−0.04	0.05	−0.15	0.07	−0.56	1	9	−0.85	0.4189	
Sex	−0.15	0.22	−0.6	0.3	−0.16	1	69	−0.68	0.4956	
Litter									0.1866	

**Table 12:** Results of Mann–Whitney U tests comparing the DPQ personality factors and fear of loud noises between the treatment and the control group.

Dependent Variable	U	Z	*p*
Factor 1—Fearfulness	508	−0.46	0.64
Factor 2—Aggression towards people	521	0.30	0.77
Factor 3—Activity/excitability	537.5	0.08	0.93
Factor 4—Trainability	424.5	1.53	0.13
Factor 5—Aggression towards animals	523.5	−0.26	0.79
Fear of loud noises (individual question)	454	−1.15	0.25

**Table 13:** Results of Kruskal–Wallis tests assessing the effect of litter on the DPQ personality factors and fear of loud noises, and the corrected alpha level following sequential Bonferroni correction.

Dependent Variable	H	*p*	Bonferroni-Corrected α
Factor 1—Fearfulness	24.3	0.0069	0.0167
Factor 2—Aggression towards people	27.35	0.0023	0.0083
Factor 3—Activity/excitability	7.2	0.7069	0.05
Factor 4—Trainability	12.19	0.2724	0.025
Factor 5—Aggression towards animals	17.13	0.0716	0.0167
Fear of loud noises (individual question)	22.39	0.0132	0.0125

## Data Availability

Data are available from the corresponding author upon reasonable request.

## Supplementary Materials

The following supporting information can be downloaded at: https://www.mdpi.com/article/10.3390/ani12223067/s1, Table S1: Demographic details of the subjects.

## Appendix A

Factors: facets and individual questions of the Dog Personality Questionnaire (short form [50]; German version validated by [52]).

**Table:**

**Factor 1—Fearfulness**		
	**Facet 1—Fear of People**	
	1 *	Dog is relaxed when greeting people.
	6	Dog is shy.
	27	Dog behaves fearfully towards unfamiliar people.
	**Facet 2—Nonsocial Fear**	
	3	Dog is anxious.
	11 *	Dog is confident.
	22*	Dog adapts easily to new situations and environments.
	**Facet 3—Fear of Dogs**	
	13	Dog avoids other dogs.
	21	Dog behaves submissively (e.g., rolls over, avoids eye contact, licks lips) when greeting other dogs.
	42	Dog behaves fearfully towards other dogs.
	**Facet 4—Fear of Handling**	
	16	Dog behaves fearfully during visits to the veterinarian.
	35	Dog exhibits fearful behaviours when restrained.
	44	Dog behaves fearfully when groomed (e.g., nails trimmed, brushed, bathed, ears cleaned).
**Factor 2—Aggression towards People**		
	**Facet 1—General Aggression**	
	7	Dog behaves aggressively towards unfamiliar people.
	18 *	Dog is friendly towards unfamiliar people.
	40	Dog shows aggression when nervous or fearful.
	**Facet 2—Situational Aggression**	
	25	Dog behaves aggressively in response to perceived threats from people (e.g., being cornered, having collar reached for).
	30	Dog behaves aggressively during visits to the veterinarian.
	36	Dog aggressively guards coveted items (e.g., stolen item, treats, food bowl).
**Factor 3—Activity/Excitability**		
	**Facet 1—Excitability**	
	15	Dog is boisterous.
	31	Dog seeks constant activity.
	41 *	Dog tends to be calm.
	**Facet 2—Playfulness**	
	9 *	Dog gets bored in play quickly.
	17	Dog enjoys playing with toys.
	33	Dog retrieves objects (e.g., balls, toys, sticks).
	**Facet 3—Active Engagement**	
	4 *	Dog is lethargic.
	14	Dog works at tasks (e.g., getting treats out of a Kong, shredding toys) until entirely finished.
	24	Dog is curious.
	**Facet 4—Companionability**	
	20	Dog seeks companionship from people.
	26 *	Dog is aloof.
	37	Dog is affectionate.
**Factor 4—Responsiveness to Training**		
	**Facet 1—Trainability**	
	29 *	Dog is slow to respond to corrections.
	38 *	Dog ignores commands.
	43	Dog is able to focus on a task in a distracting situation (e.g., loud or busy places, around other dogs).
	**Facet 2—Controllability**	
	5	When off leash, dog comes immediately when called.
	10 *	Dog is quick to sneak out through open doors; gates.
	32	Dog leaves food or objects alone when told to do so.
**Factor 5—Aggression towards Animals**		
	**Facet 1—Aggression towards Dogs**	
	2	Dog behaves aggressively toward dogs.
	19 *	Dog is playful with other dogs.
	34 *	Dog is friendly towards other dogs.
	**Facet 2—Prey Drive**	
	8	Dog likes to chase squirrels, birds, or other small animals.
	23	Dog likes to chase bicycles, joggers, and skateboarders.
	39	Dog behaves aggressively towards cats.
	**Facet 3—Dominance over other Dogs**	
	12	Dog is dominant over other dogs.
	28 *	Dog willingly shares toys with other dogs.
	45	Dog is assertive or pushy with other dogs (e.g., if in a home with other dogs, when greeting).

Note. Items marked with an asterisk are reverse coded items. Additional question: Dog is afraid of loud noises.
