# Supplementary material for: Optimising Puppy Socialisation–Short- and Long-Term Effects of a Training Programme during the Early Socialisation Period
Source: Animals (Basel). 2022 Nov 8;12(22):3067. doi: 10.3390/ani12223067 (PMC9687081; doi:10.3390/ani12223067)
Supplement: Supplementary file 1 [file animals-12-03067-s001.zip › animals-1956444-supplementary.pdf]

Table S1. Demographic details of the subjects.

Nr.	Litter	ID	Sex	Treatment	Breed	Age at test (days)
1	Sheltie1	Sheltie1 1	female	control	Shetland Sheepdog	43
2	Sheltie1	Sheltie1 2	female	control	Shetland Sheepdog	43
3	Sheltie1	Sheltie1 3	male	training	Shetland Sheepdog	43
4	Sheltie1	Sheltie1 4	female	training	Shetland Sheepdog	43
5	Sheltie2	Sheltie2 1	female	training	Shetland Sheepdog	51
6	Sheltie2	Sheltie2 2	male	control	Shetland Sheepdog	51
7	Sheltie2	Sheltie2 3	male	training	Shetland Sheepdog	51
8	Sheltie2	Sheltie2 4	male	training	Shetland Sheepdog	51
9	Sheltie2	Sheltie2 5	male	control	Shetland Sheepdog	51
10	MiniAussie1	MiniAussie1 1	male	control	Mini Australian Shep-herd	41
11	MiniAussie1	MiniAussie1 2	female	training	Mini Australian Shep-herd	41
12	MiniAussie1	MiniAussie1 3	female	control	Mini Australian Shep-herd	41
13	MiniAussie1	MiniAussie1 4	male	control	Mini Australian Shep-herd	41
14	MiniAussie1	MiniAussie1 5	female	training	Mini Australian Shep-herd	41
15	MiniAussie1	MiniAussie1 6	female	control	Mini Australian Shep-herd	41
16	MiniAussie1	MiniAussie1 7	male	training	Mini Australian Shep-herd	41
17	Aussie1	Aussie1 1	female	control	Australian Shepherd	40
18	Aussie1	Aussie1 2	female	training	Australian Shepherd	40
19	Aussie1	Aussie1 3	female	training	Australian Shepherd	40
20	Aussie1	Aussie1 4	male	control	Australian Shepherd	40
21	Aussie2	Aussie2 1	female	control	Australian Shepherd	46
22	Aussie2	Aussie2 2	female	control	Australian Shepherd	46
23	Aussie2	Aussie2 3	female	control	Australian Shepherd	46
24	Aussie2	Aussie2 4	female	training	Australian Shepherd	46

25	Aussie2	Aussie2 5	female	training	Australian Shepherd	46
26	Aussie2	Aussie2 6	female	training	Australian Shepherd	46
27	Aussie2	Aussie2 7	female	training	Australian Shepherd	46
28	Aussie3	Aussie3 1	male	control	Australian Shepherd	46
29	Aussie3	Aussie3 2	female	training	Australian Shepherd	46
30	Aussie3	Aussie3 3	male	training	Australian Shepherd	46
31	Aussie3	Aussie3 4	female	control	Australian Shepherd	46
32	Aussie3	Aussie3 5	female	control	Australian Shepherd	46
33	Aussie3	Aussie3 6	male	control	Australian Shepherd	46
34	Aussie3	Aussie3 7	female	training	Australian Shepherd	46
35	Aussie3	Aussie3 8	male	training	Australian Shepherd	46
36	Aussie4	Aussie4 1	female	training	Australian Shepherd	48
37	Aussie4	Aussie4 2	female	control	Australian Shepherd	48
38	Aussie4	Aussie4 3	male	training	Australian Shepherd	48
39	Aussie4	Aussie4 4	male	control	Australian Shepherd	48
40	Aussie4	Aussie4 5	female	control	Australian Shepherd	48
41	Aussie4	Aussie4 6	male	control	Australian Shepherd	48
42	Aussie4	Aussie4 7	male	training	Australian Shepherd	48
43	Labrador1	Labrador1 1	female	control	Labrador Retriever	41
44	Labrador1	Labrador1 2	male	training	Labrador Retriever	41
45	Labrador1	Labrador1 3	male	control	Labrador Retriever	41
46	Labrador1	Labrador1 4	male	control	Labrador Retriever	41
47	Labrador1	Labrador1 5	female	training	Labrador Retriever	41
48	Labrador1	Labrador1 6	female	training	Labrador Retriever	41
49	Labrador1	Labrador1 7	male	training	Labrador Retriever	41
50	Labrador1	Labrador1 8	female	control	Labrador Retriever	41
51	Labrador1	Labrador1 9	female	training	Labrador Retriever	41
52	Herder1	Herder1 1	female	control	Dutch Shepherd	41
53	Herder1	Herder1 2	female	control	Dutch Shepherd	41
54	Herder1	Herder1 3	female	training	Dutch Shepherd	41
55	Herder1	Herder1 4	male	training	Dutch Shepherd	41

56	Herder1	Herder1 5	male	training	Dutch Shepherd	41
57	Herder1	Herder1 6	female	control	Dutch Shepherd	41
58	Herder1	Herder1 7	female	training	Dutch Shepherd	41
59	Herder1	Herder1 8	male	control	Dutch Shepherd	41
60	Pitbull1	Pitbull1 1	female	training	American Pitbull Terrier	41
61	Pitbull1	Pitbull1 2	male	training	American Pitbull Terrier	41
62	Pitbull1	Pitbull1 3	male	training	American Pitbull Terrier	41
63	Pitbull1	Pitbull1 4	female	control	American Pitbull Terrier	41
64	Pitbull1	Pitbull1 5	male	control	American Pitbull Terrier	41
65	Pitbull1	Pitbull1 6	male	control	American Pitbull Terrier	41
66	Pitbull1	Pitbull1 7	female	control	American Pitbull Terrier	41
67	Pitbull1	Pitbull1 8	female	control	American Pitbull Terrier	41
68	Pitbull1	Pitbull1 9	male	control	American Pitbull Terrier	41
69	Pitbull1	Pitbull1 10	male	training	American Pitbull Terrier	41
70	Pitbull1	Pitbull1 11	female	training	American Pitbull Terrier	41
71	Icelandic1	Icelandic1 1	female	training	Icelandic Sheepdog	42
72	Icelandic1	Icelandic1 2	male	control	Icelandic Sheepdog	42
73	Icelandic1	Icelandic1 3	female	control	Icelandic Sheepdog	42
74	Icelandic1	Icelandic1 4	male	training	Icelandic Sheepdog	42
75	Icelandic1	Icelandic1 5	male	training	Icelandic Sheepdog	42
76	Icelandic1	Icelandic1 6	female	control	Icelandic Sheepdog	42
77	Setter1	Setter1 1	female	training	English Setter	43
78	Setter1	Setter1 2	male	control	English Setter	43

79	Setter1	Setter1 3	female	training	English Setter	43
80	Setter1	Setter1 4	male	training	English Setter	43
81	Setter1	Setter1 5	female	control	English Setter	43
82	Setter1	Setter1 6	female	control	English Setter	43
83	Setter1	Setter1 7	female	training	English Setter	43
